# Supplementary material for: Hypersensitive detection of single millimeter vascular emboli from adhesive in vivo
Source: Nat Commun. 2026 Feb 10;17:1823. doi: 10.1038/s41467-026-68534-w (PMC12921325; doi:10.1038/s41467-026-68534-w)
Supplement: Supplementary file 1 — Supplementary Information [file 41467_2026_68534_MOESM1_ESM.pdf]

Supplementary Information

Hypersensitive detection of single millimeter vascular emboli from adhesive *in vivo*

Ruihan Liu[1,†], Shuo Li[1,†], Xingyu Gao[1,†], Quan Zou[1], Gang Shu[2], Cai Zhang[2], Jinbin Pan[3], Xiaoyuan Chen[4,*] & Shao-Kai Sun[1,*]

[1]School of Medical Imaging, Division of Medical Technology, Tianjin Key Laboratory of Functional Imaging, Tianjin Medical University, Tianjin, China.

[2]Department of Radiology, Tianjin Medical University Cancer Institute and Hospital, National Clinical Research Centre of Cancer, Tianjin Clinical Research Center for Cancer, Tianjin, China.

[3]Department of Radiology, Tianjin Key Laboratory of Functional Imaging, Tianjin Medical University General Hospital, Tianjin, China.

[4]Shandong Provincial Key Laboratory of Precision Oncology, Shandong Cancer Hospital and Institute, Shandong First Medical University and Shandong Academy of Medical Sciences, Jinan, China.

[†]These authors contributed equally: Ruihan Liu, Shuo Li, Xingyu Gao.

*Corresponding authors. Email: chen9647@gmail.com (Xiaoyuan Chen); shaokaisun@tmu.edu.cn (Shao-Kai Sun)

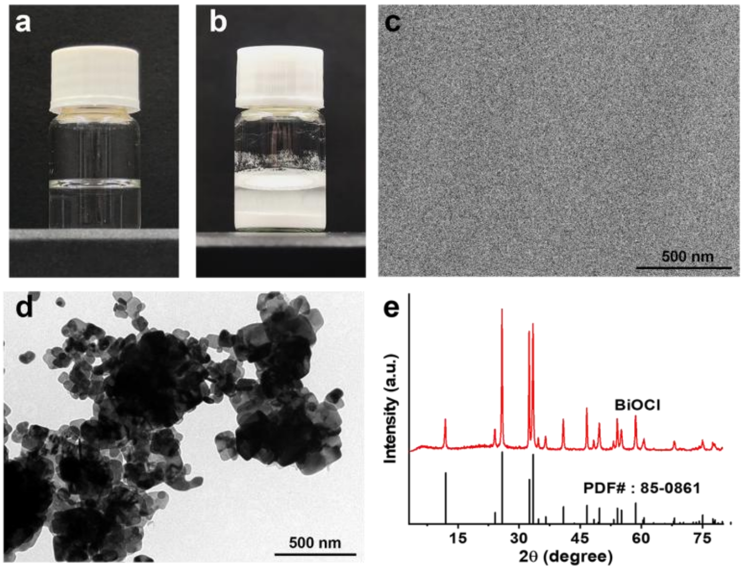

Fig. S1. Photographs of Bi-DTPA (**a**) and BiOCl (**b**) dispersions in water under static conditions. TEM images of Bi-DTPA (**c**) and BiOCl (**d**). (**e**) XRD pattern of BiOCl.

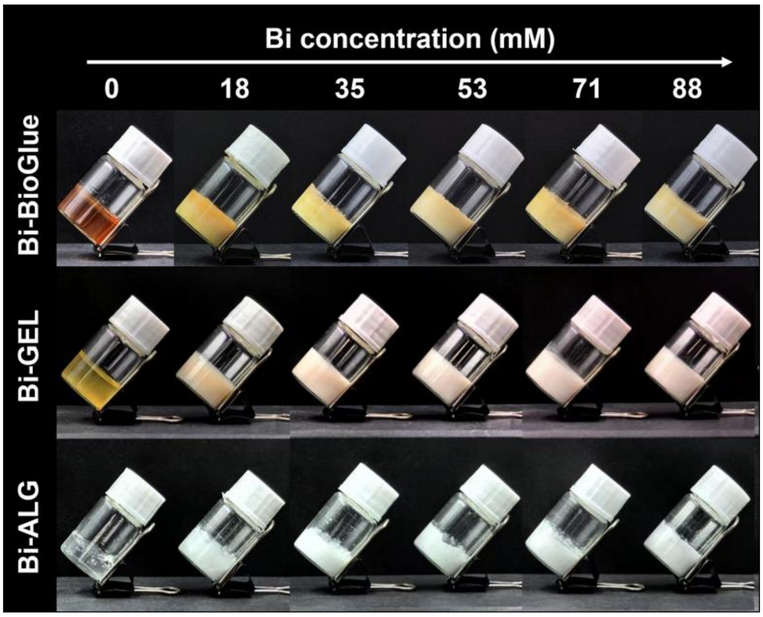

Fig. S2. Photographs of BiOCl-doped BioGlue, gelatin, and sodium alginate with different concentrations (0-88 mM) of BiOCl.

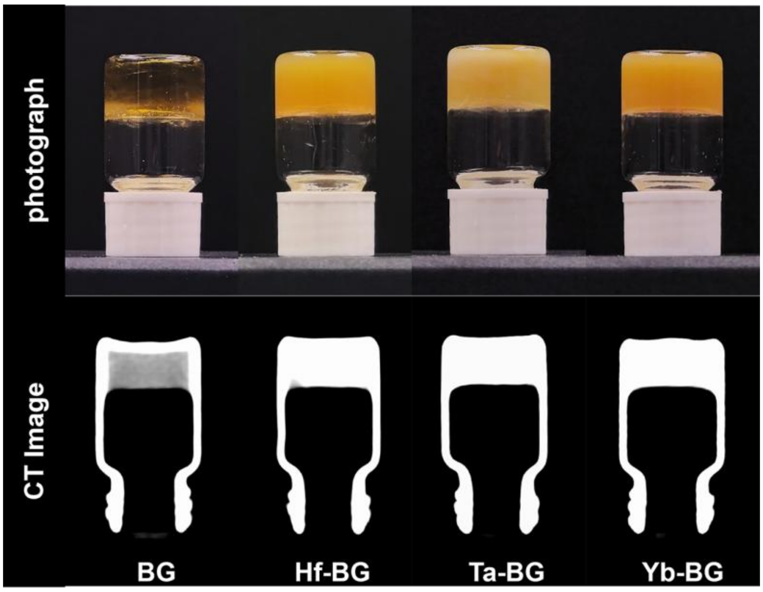

Fig. S3. Photographs and corresponding CT images of conventional BioGlue, Hf-BioGlue, Ta-BioGlue and Yb-BioGlue with the Hf, Ta, and Yb concentration of 105 mM.

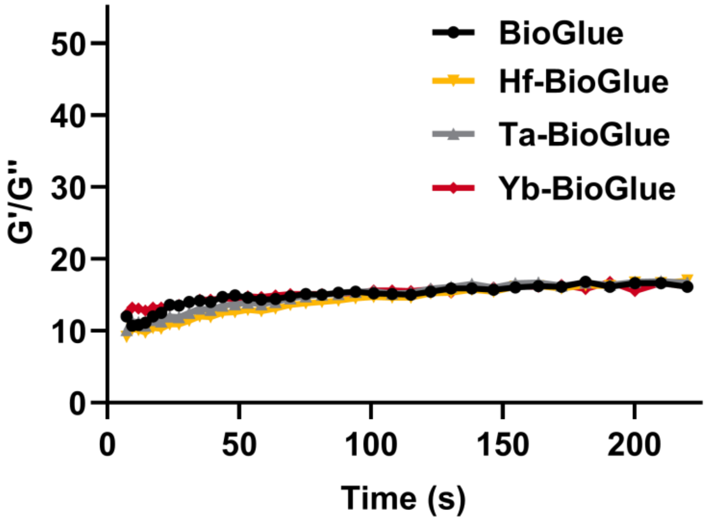

Fig. S4. Dynamic oscillatory time sweep measurements of BioGlue, Ta-BioGlue, Hf-BioGlue and Yb-BioGlue.

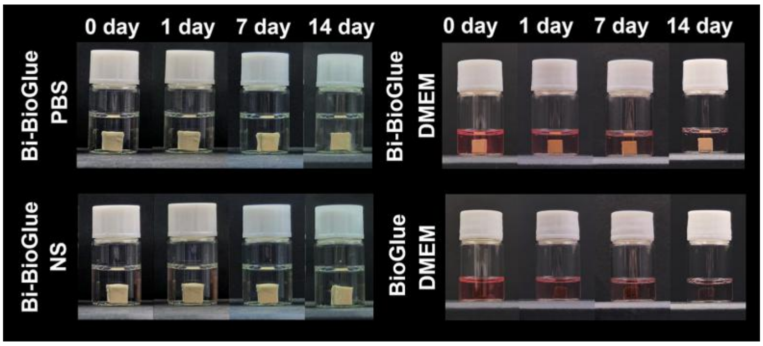

Fig. S5. Photographs of Bi-BioGlue immersed in PBS, normal saline and DMEM for 14 days. Photographs BioGlue immersed in DMEM for 14 days.

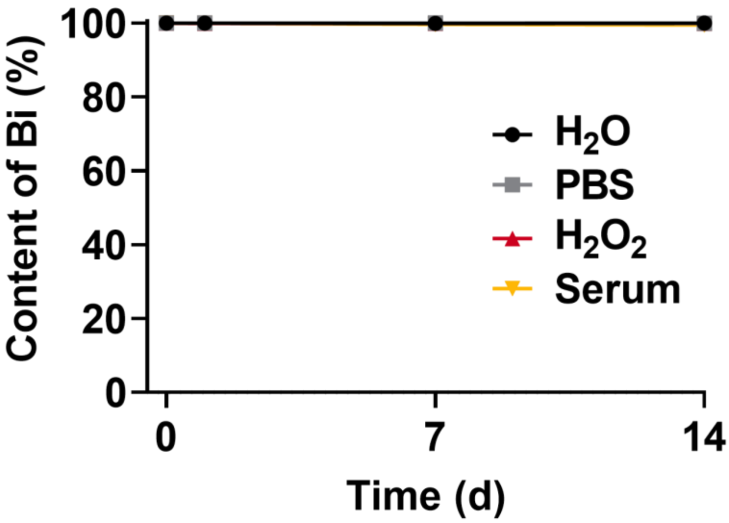

Fig. S6. The release curves of Bi elements in Bi-BioGlue within 14 days across different media including H_2O, PBS, H_2O_2, and serum (n = 3 independent samples; data shown as mean ± SD).

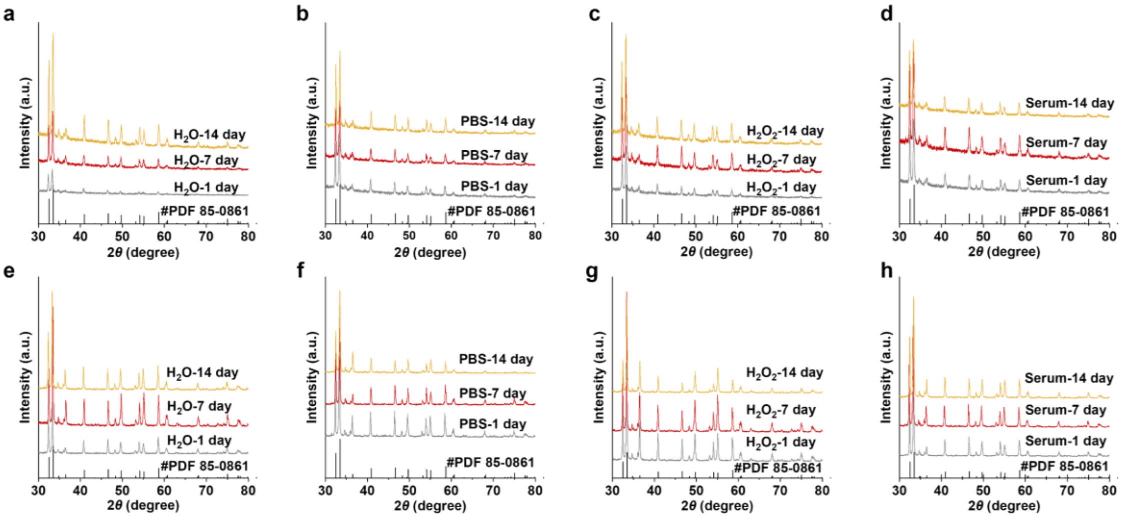

Fig. S7. XRD patterns of Bi-BioGlue after incubation in different media: H_2O (**a**), PBS (**b**), H_2O_2 (**c**), and serum (**d**) on days 1, 7, and 14. XRD patterns of BiOCl after incubation in different dispersion systems: H_2O (**e**), PBS (**f**), H_2O_2 (**g**), and serum (**h**) on days 1, 7, and 14.

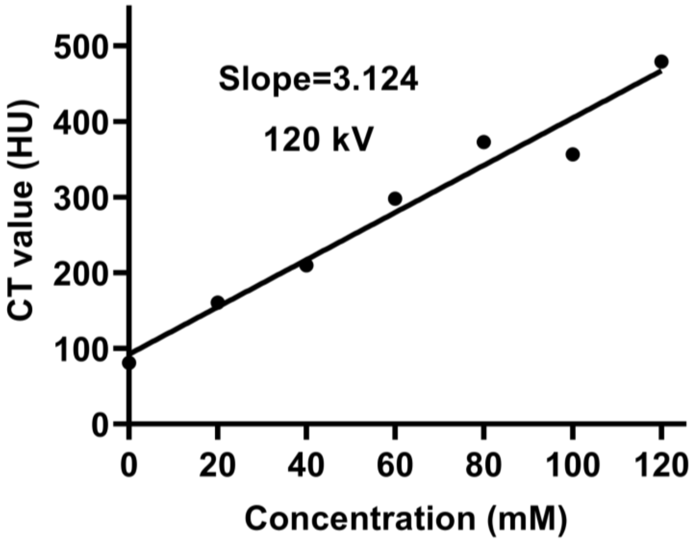

Fig. S8. CT values of I-BioGlue at different concentrations of I (n = 3 technical replicates; data shown as mean ± SD).

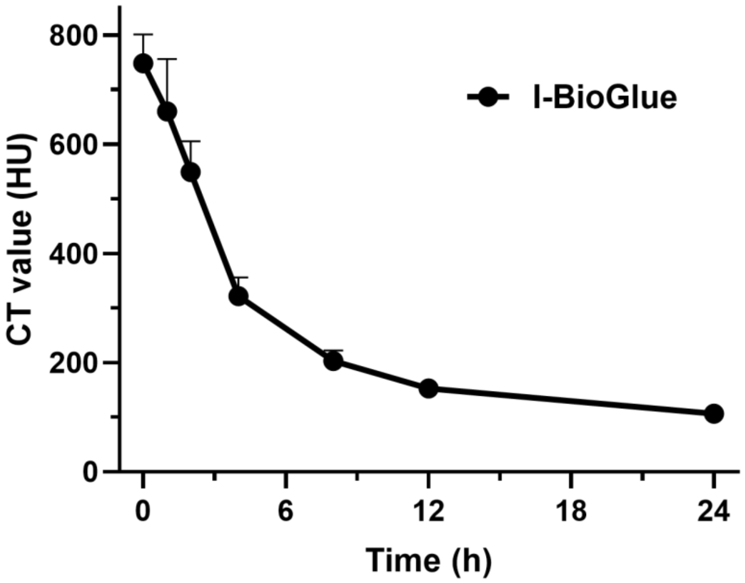

Fig. S9. CT value changes over time for I-BioGlue in water for 24 h (n = 3 technical replicates; data shown as mean ± SD).

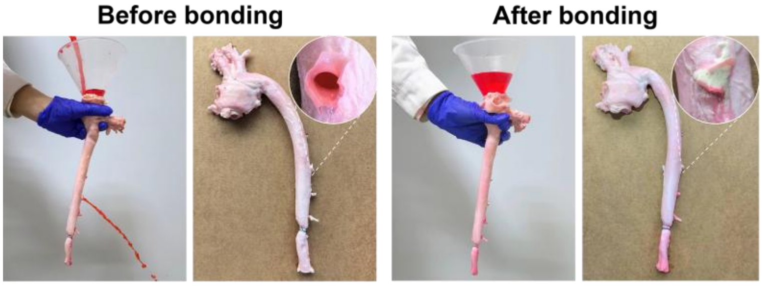

Fig. S10. Photographs of porcine aortic vessels infused with red ink before and after bonding with Bi-BioGlue.

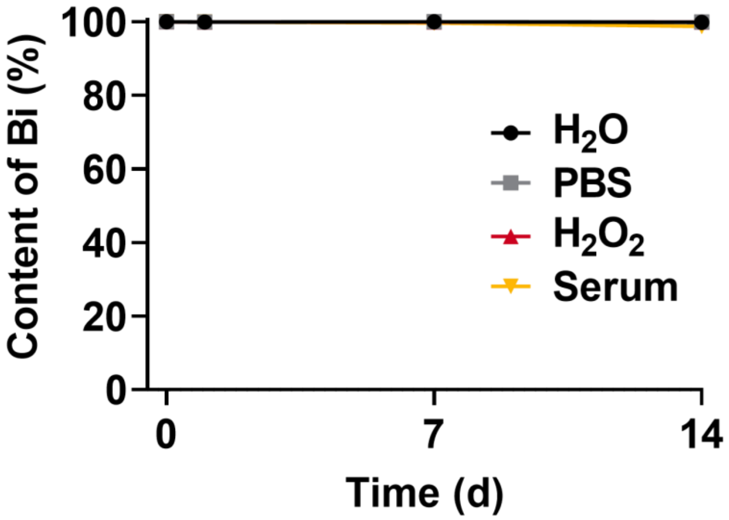

Fig. S11. The release curves of Bi elements in BiOCl within 14 days across different media including H_2O, PBS, H_2O_2, and serum (n = 3 independent samples; data shown as mean ± SD).

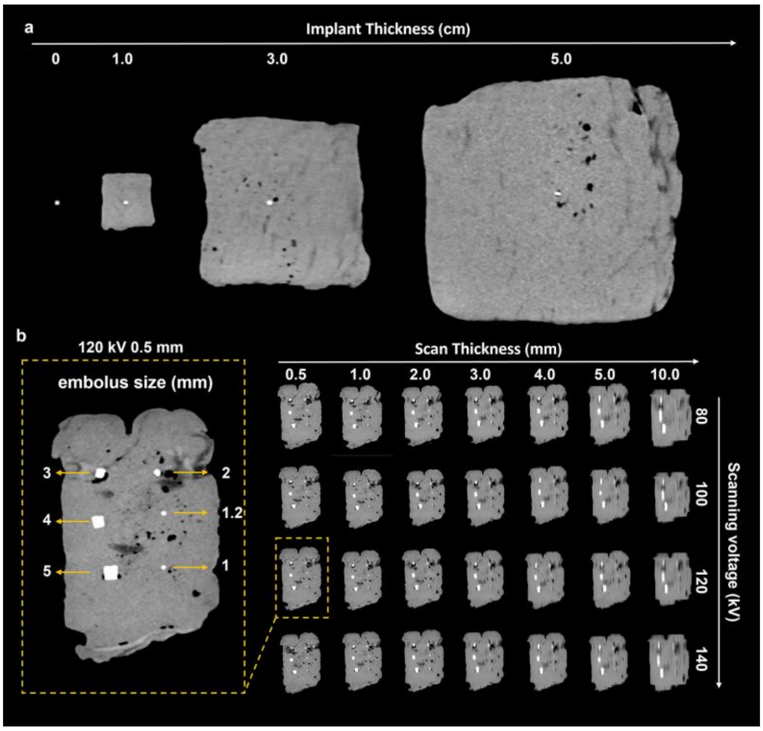

Fig. S12. (**a**) CT images of Bi-BioGlue emboli in red meat with different thicknesses (0, 1.0, 3.0, 5.0 cm). (**b**) CT images of Bi-BioGlue emboli of different sizes in red meat under different voltages (80, 100, 120, 140 kV) and scanning layer thicknesses (0.5, 1.0, 2.0, 3.0, 4.0, 5.0, 10.0 mm).

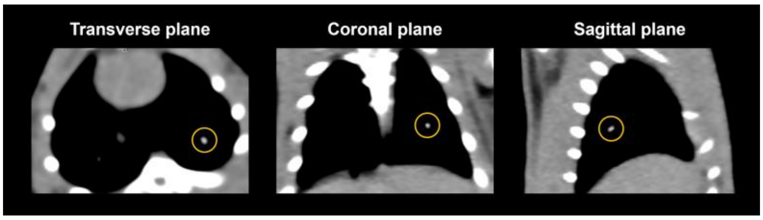

Fig. S13. CT images of 1.0 mm Bi-BioGlue embolus *in vivo*.

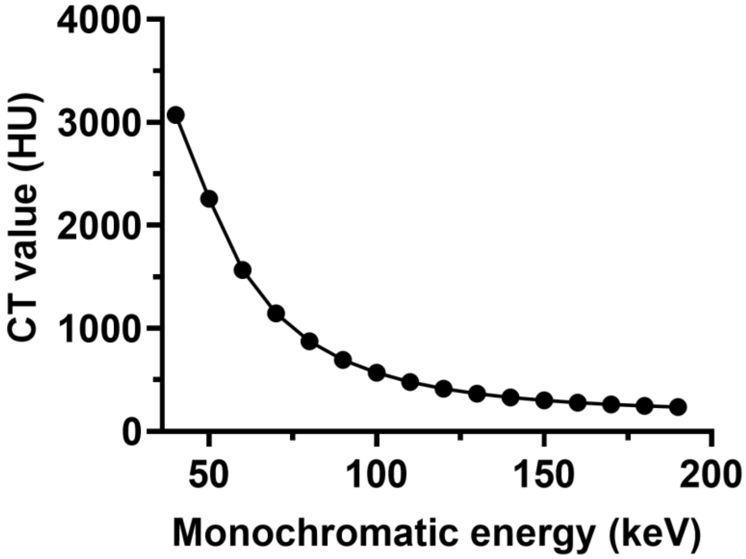

Fig. S14. Spectral CT values of Iohexol at different monochromatic energies (n = 3 technical replicates; data shown as mean ± SD).

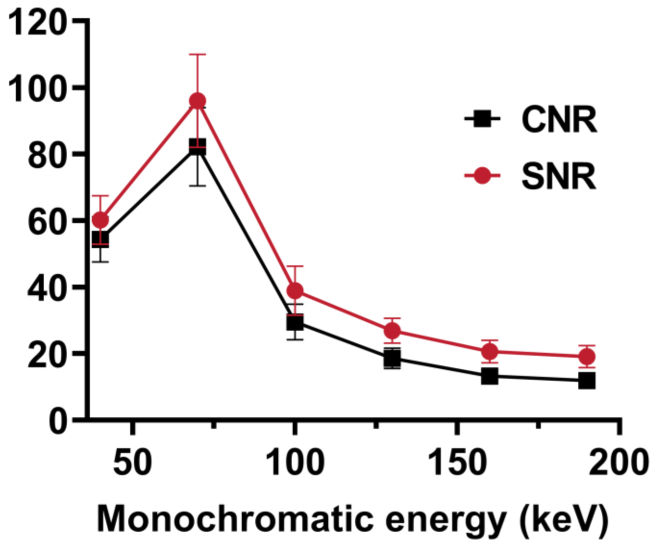

Fig. S15. The SNR and CNR curves of Bi-BioGlue-induced embolus at different monochromatic energies *in vivo* (n = 3 technical replicates; data shown as mean ± SD).

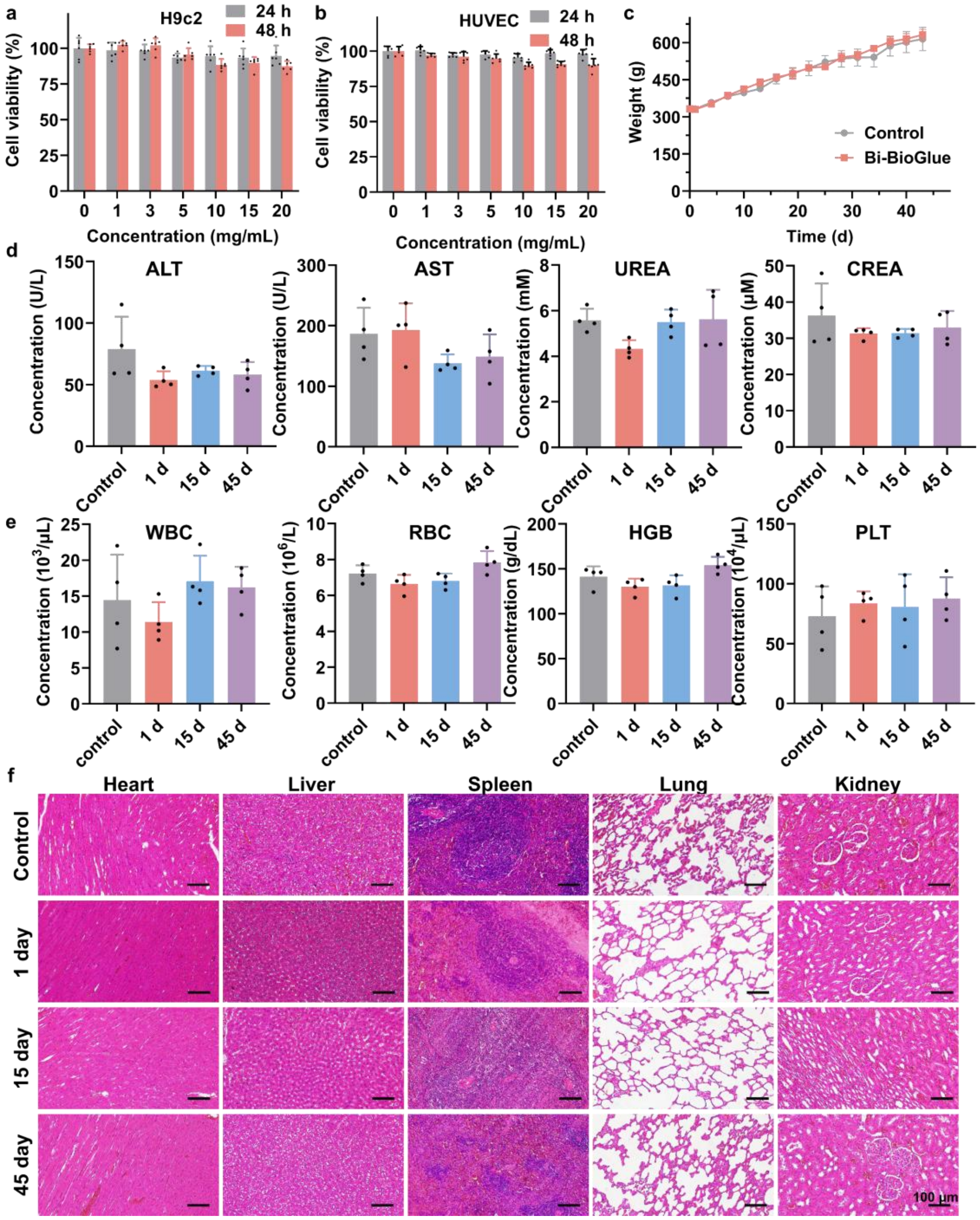

Fig. S16. Biosafety evaluation of Bi-BioGlue. Cell viabilities of H9c2 cells (**a**) and HUVEC cells (**b**) after incubated with different concentrations of Bi-BioGlue for 24 or 48 h (n = 6 biological experiments; mean ± SD). (**c**) Body weight changes of control group and Bi-BioGlue group in 45 days (n = 4 rats per group; data shown as mean ± SD). Biochemical analysis (**d**) and routine blood tests (**e**) of control group, and Bi-BioGlue group in 45 days (n = 4 rats per group; data shown as mean ± SD). (**f**) H&E staining images of major organs (heart, liver, spleen, lung, and kidney) collected from the rats in control group and Bi-BioGlue groups.
